# Injectable Hydrogel for NIR-II Photo-Thermal Tumor Therapy and Dihydroartemisinin-Mediated Chemodynamic Therapy

**DOI:** 10.3389/fchem.2020.00251

**Published:** 2020-04-07

**Authors:** Danyang Chen, Chuang Chen, Chunyu Huang, Tongkai Chen, Zeming Liu

**Affiliations:** ^1^Department of Plastic Surgery, Zhongnan Hospital of Wuhan University, Wuhan, China; ^2^Department of Breast and Thyroid Surgery, Renmin Hospital of Wuhan University, Wuhan, China; ^3^Science and Technology Innovation Center, Guangzhou University of Chinese Medicine, Guangzhou, China

**Keywords:** injectable hydrogel, NIR-II photothemal therapy, dihydroartemisinin, Hu Kaiwen ink, chemodynamic therapy

## Abstract

In traditional Chinese medicine, dihydroartemisinin (DHA) is the focus of extensive attention because of its unique activity with Fe^2+^ to produce reactive oxygen species (ROS) and promote apoptosis. In this work, we designed a newfangled ink@hydrogel containing FeCl_3_, traditional Chinese ink (Hu Kaiwen ink), and agarose hydrogel to create a synergistic activity with DHA in the treatment of cancer. When the system is irradiated under 1,064 nm for a few minutes, the ink in the ink@hydrogel converts the light to heat and hyperthermia causes the reversible hydrolysis of hydrogel. Then, Fe^3+^ quickly diffuses from the hydrogel to the tumor microenvironment and is reduced to Fe^2+^ to break the endoperoxide bridge in pre-injected DHA, which results in the release of free radicals for a potent anticancer action. To our knowledge, this is the first report of a hydrogel tumor therapy system that induces a photo-thermal response in the second near infrared window (NIR-II). *in vivo* experiments also showed a significant effect of DHA-Fe^2+^ in chemodynamic therapy (CDT) and in photo-thermal therapy. This hydrogel platform provided an encouraging idea for synergistic tumor therapy.

## Introduction

Cancer is the leading cause of death worldwide and poses a huge threat to human health, even after the recent significant research advances (Li et al., 2017; Wu et al., 2018; Zhang et al., 2019). Recently, new tumor treatments, such as photothermal therapy (PTT) (Chu and Dupuy, 2014; Song et al., 2015; Sun et al., 2017; Yang et al., 2017; Jiang et al., 2018; Zhou et al., 2018; Liu et al., 2019) and chemodynamic therapy (CDT) (Jia et al., 2016), have attracted much attention due to the limited side effects and drug resistance compared with traditional strategies like chemotherapy, surgery, and radiotherapy.

The emerging PTT treatment uses nanoparticles as photo-thermal agents (PTAs) (Sun et al., 2017; Jiang et al., 2018; Cao et al., 2019; Yang et al., 2019) since they have a high absorbance in the near-infrared (NIR) to convert light to thermal energy and induce tumor ablation. PTT causes little damage to the patient and has minimal side effects. It can be used by itself or combined with other therapies like photodynamic therapy (PDT) (Hu et al., 2019; Liang et al., 2019). However, most PTAs have a limited penetration depth since they are only active in the NIR-I window (750–1,000 nm), which reduces their efficiency and clinical performance. Although NIR-II radiation (1,000–1,350 nm) (Lin et al., 2017; Yu et al., 2017; Cao et al., 2019) has a better maximum permissible exposure (MPE) and a larger penetration depth, there is a lack of PTAs with a strong enough absorption and a high enough photo-thermal conversion efficiency. Therefore, it is necessary to explore PTAs that are active in the NIR-II window for tumor therapy. Traditional Chinese ink like the Hu Kaiwen ink has a good photo-thermal conversion efficiency, a high stability in liquid, and an excellent biocompatibility. It is therefore expected to have a great potential as an NIR-II photo-thermal material (Wang et al., 2017; Ouyang et al., 2019). Recently, light-responsive hydrogel (Xing et al., 2016; Niu et al., 2017; Hou et al., 2018; Qiu et al., 2018; Wu et al., 2019) was introduced as creative and novel drug release vessel for tumor treatment. It has attached much attention and has a great potential for the controlled release of active agents. Furthermore, the drug release rate can be controlled by changing the power density of the incident light and the exposure time to modify the dissolution of the hydrogel. However, the photo-thermal response of hydrogels in the NIR-II spectrum has not yet been studied.

In traditional Chinese medicine, dihydroartemisinin (DHA) and its derivatives have been extensively used as an effective anti-malaria drug since the 1970s (Wang et al., 2016). Recently, they have been studied as alternative tumor therapeutic agents to kill various tumor cells *in vitro* and *in vivo* through the generation of active oxygen radicals via the homolytic cleavage of the weak endoperoxide bridge accelerated by high concentrations of ferrous irons (Wang et al., 2016). However, the insufficient availability of Fe^2+^ in the tumor tissues severely limits the clinical performance and an urgent solution is needed to increase the Fe^2+^ content in the tumor tissues and create a synergetic therapy with DHA.

Herein, we designed a newfangled ink@hydrogel containing FeCl_3_, traditional Chinese ink (Hu Kaiwen ink), and agarose hydrogel to act synergistically with DHA in the treatment of cancer. When the system is irradiated at 1,064 nm for a few minutes, the ink in the ink@hydrogel converts light to heat and hyperthermia causes the reversible hydrolysis of hydrogel. Then, Fe^3+^ ions diffuse from the hydrogel to the tumor microenvironment and are reduced to Fe^2+^ to promote the breakage of the endoperoxide bridge in the pre-injected DHA. This results in the release of free radicals for a potent anticancer effect. To our best knowledge, this is the first report of a hydrogel system for tumor therapy that creates a photo-thermal response in the NIR-II biological window. *In vivo* experiments are carried out to determine the efficiency of DHA-Fe^2+^ in chemodynamic therapy (CDT) and in photo-thermal therapy. This special hydrogel treatment way provided a great idea for synergistic tumor therapy.

## Results and Discussion

### Synthesis and Characterization of the Hydrogel

**Figure 1:**
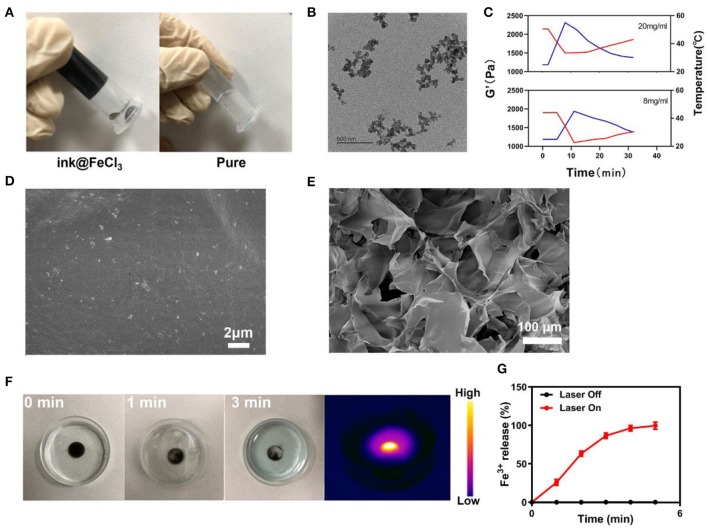
**(A)** Various hydrogels prepared. **(B)** TEM image of the ink. **(C)** Rheological curve (red line) and corresponding temperature curve (blue line) of ink@hydrogel under NIR irradiation at 1 W/cm^2^. **(D)** SEM image of the ink@hydrogel. **(E)** SEM image of the ink@hydrogel at a lower magnification. **(F)** Hydrogel dissolved by the 1,064-nm laser irradiation (1 W/cm^2^). **(G)** Release rate of Fe^3+^ from the ink@hydrogel with (red) and without (black) laser irradiation.

First, the ink was diluted to a light concentration to produce a usable sample. Figure 1A show the various hydrogels prepared. Each hydrogel was prepared in a centrifuge tube and did not flow downwards once gelation was complete. The nanoscale morphology of the ink was determined by transmission electron microscopy (TEM) (Figure 1B). The ink mostly presented small aggregates. Rheology measurements on the ink@hydrogel (a mixture of agarose hydrogel and ink) with different ink concentrations showed a decrease in the storage modulus for increasing ink concentrations, as shown in Figure 1C. When the temperature increases, the storage modulus of the hydrogel decreases, which confirms the successful formation of the hydrogel. Representative SEM (scanning electron microscope) images of the ink@hydrogel (Figures 1D,E) indicated a complex pore size distribution where different concentrations and temperatures produced different pore sizes. A power density of 1 W/cm^2^ at 1,064 nm irradiation was used to evaluate the temperature control ability of ink@hydrogel (Figure 1F). Initially, the dark colors of conglomerated ink@hydrogel were observed, but persistent laser irradiation faded the colors, indicating the degradation of the ink@hydrogel. Infrared thermal imaging (Figure 1F) also confirmed the increase of the temperature in the ink@hydrogel upon laser irradiation. We measured the release rate of Fe^3+^ in the hydrogel with or without laser irradiation (Figure 1G). The ink@hydrogel gradually dissolved and released Fe^3+^ under laser irradiation at 1,064 nm (1 W/cm^2^), whereas there was no significant change in the group without laser irradiation, which showed that the hydrogel was irradiated by laser irradiation to dissolve and release iron ions.

### Photo-Thermal of the ink@hydrogel for PTT

**Figure 2:**
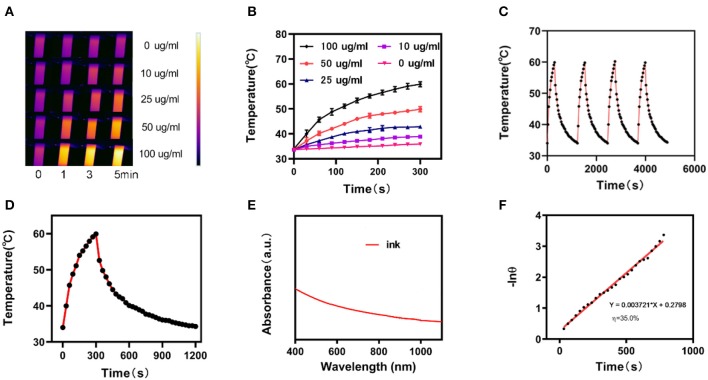
**(A)** Infrared thermographic maps of ink solution in the centrifuge tube upon NIR laser irradiation for 0–5 min. **(B)** Temperature increase for the different ink concentrations upon laser irradiation at 1,064 nm and 1 W/cm^2^ for 5 min. **(C)** Temperature variation of an ink solution at 100 mg/mL under cyclic laser irradiation during which the laser is on for 20 min in each cycle. **(D)** Temperature profile of an ink solution at 100 mg/mL upon heating when the laser is on and subsequent cooling once the laser is turned off. **(E)** UV-visible-NIR absorbance spectrum of an ink solution. **(F)** Calculation of the time constant for the heat transfer using a linear regression of the cooling profile.

The photo-thermal performance of the ink was estimated by irradiating a centrifuge tube containing an aqueous ink dispersion at various concentrations (0, 10, 25, 50, and 100 μg/mL) with an NIR laser (1,064 nm, 1 W/cm^2^) in parallel, while capturing the infrared thermal images of the ink solutions to confirm the temperature response during irradiation (Figures 2A,B). The photo-thermal heating effect of the ink was concentration-dependent for a fixed irradiation power. Higher ink concentrations resulted in a greater heating effect, which indicated that the ink efficiently converted light to thermal energy. Furthermore, the temperature of the ink solution at 100 μg/mL increased from the initial 35°C to nearly 60°C in 5 min. This suggests that the laser irradiation triggers a forceful hyperthermia and the elevated temperature is sufficient to damage tumor cells through the destruction of the intracellular protein and genetic materials. The ink solution was irradiated at 1,064 nm with 1 W/cm^2^ for 5 min. Then, the laser was turned off to allow the initial temperature to recover. This cycle was repeated four times (Figure 2C) to demonstrate that the variation of the peak temperature in every cycle was negligible and that the photo-thermal performance of the ink was stable and reproducible during cycling. The photo-thermal conversion efficiency (η) of the ink was calculated from the data of Figures 2D,F and was as high as 35.0%, which is higher than Au nanorods (21%), graphene quantum dots (28.58%), and Ti_3_C_2_ nanosheets (30.6%) (Liu et al., 2015; Rasool et al., 2016; Shao et al., 2016; Deng et al., 2019). Figure 2E shows the UV-visible-NIR absorbance spectrum of the ink solution, revealing a broad and strong absorbance between 800 and 1,100 nm, without any obvious peak. This indicates that the hydrogel is a very suitable photo-thermal material.

### *In vitro* Combination Therapy

**Figure 3:**
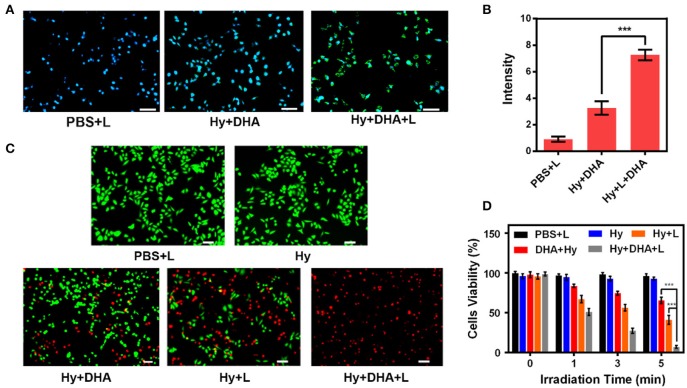
**(A)** DCFH-DA (2,7-Dichlorodi-hydrofluorescein diacetate) staining in 4T1 cells upon different treatments. Scale bar: 50 μm. **(B)** Fluorescence intensity of DCFH-DA from **(A)**. **(C)** Fluorescence images of 4T1 cells stained with FDA (live cells, green fluorescence) and PI (dead cells, red fluorescence) after incubation with different formulations. **(D)** Cell viability of 4T1 cells cultured in the presence of various formulations after laser irradiation. ***p < 0.005.

We prepared appropriate amounts of ink@hydrogel to generate ROS *in vitro*. After the 4T1 cells were treated with different hydrogels for 2 h, the ROS stress level in the cells was measured by fluorescence microscope (Figures 3A,B). Upon laser irradiation, the ink@hydrogel samples had a high ROS level, whereas the un-irradiated hydrogel samples and irradiated PBS control had lower ROS levels. This might be attributed to the dissolution of the ink@hydrogel released Fe^3+^ ions that were then locally reduced to Fe^2+^, which can be coupled with DHA. Next, we examined the fluorescence images of the 4T1 cells stained with FDA (Fluorescein diacetate) (live cells, green fluorescence) and PI (propidium iodide) (dead cells, red fluorescence) under different conditions (Figure 3C). By comparing the images of the ink@hydrogel group with or without laser irradiation, the viability of the 4T1 cells significantly decreased upon laser irradiation most likely due to the temperature increase that triggers the dissolution of the hydrogel and releases Fe^3+^ ions. The group with ink@hydrogel and DHA subjected to the laser irradiation showed a high level of apoptosis due to the generation of hydroxyl radicals from the breakage of endoperoxide bridges during the action of DHA and Fe^2+^. Figure 3D later confirmed the cell toxicity of the hydrogel with DHA upon laser irradiation. The cells incubated with hydrogel were damaged and died after 5 min of laser irradiation.

### *In vivo* Anti-tumor Study

**Figure 4:**
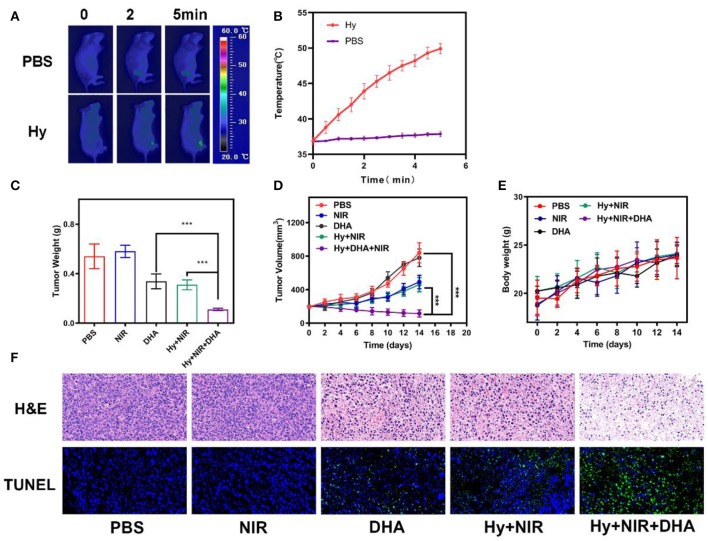
**(A)** Infrared images of the mice tissue under laser irradiation after the administration of PBS and ink@hydrogel (Hy). **(B)** Temperature of the mice upon laser irradiation. The data represent mean ± standard deviation (*n* = 3). **(C)** Evolution of the tumor weight during therapy. **(D)** Evolution of the volume of 4T1 tumors bearing female BALB/C mice after various treatments. **(E)** Body weight of nude mice recorded every other day for various treatments. **(F)** H&E and TUNEL staining of tumor sections from the 4T1 tumor-bearing mice. ***p < 0.005.

Since the *in vitro* results were very encouraging for the ink@hydrogel combined with DHA, we studied the *in vivo* potential. Mice bearing 4T1 tumors were split into five groups when the tumor volume reached ≈200 mm^3^ (*n* = 5): (1) PBS solution group, (2) laser irradiation group, (3) 5 mg/kg DHA solution group, (4) ink@hydrogel with laser irradiation, (5) ink@hydrogel and DHA with laser irradiation. The five groups significantly demonstrated the efficiency of a therapy using hydrogel combined with DHA and laser irradiation. DHA was administered by intra-peritoneal injection whereas the other solutions were administered by orthotopic injection. DHA was injected 12 h before the ink@hydrogel. Figure 4A shows an infrared image of the PBS group and the hydrogel with DHA group under laser irradiation. The temperature in the control group barely increased, whereas the *in vivo* temperature distribution in the ink@hydrogel with DHA group was raised by about 15°C in 5 min (Figure 4B). The reduced heat tolerance of the tumor tissue compared to normal cells results in the selective destruction of the tumor cells at temperatures above hyperthermia (42–47°C) (Yang et al., 2017). The volume of the tumors in each of the five groups was measured every other day using a digital caliper and the tumor weight was calculated, as shown in Figures 4C,D. Compared with the PBS and NIR irradiation group, the tumor grew slowly in the DHA group and the ink@hydrogel without DHA group under laser irradiation. The volume and the weight of the tumor in the hydrogel with DHA group were significantly lowered as the average mice weight in hydrogel with DHA group were only 0.11 g. The body-weight regularly increased in all groups during the whole therapy (Figure 4E), which confirmed that these treatments produced negligible adverse effects on the mice. We also examined the micrographs of tumor tissues stained with H&E and TUNEL (Figure 4F). The combination of the photo-thermal therapy with DHA yielded the highest apoptosis rate for the tumor cells.

### Histological Analysis

**Figure 5:**
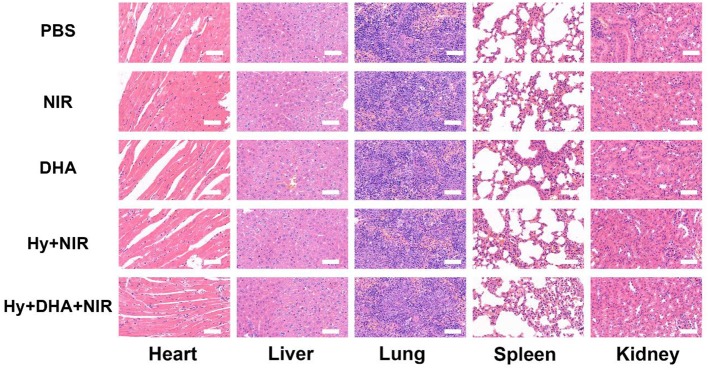
Evaluation of the toxicity *in vivo*. Histological data (H&E staining) obtained in the major organs (heart, liver, spleen, lung, and kidney) of the mice 14 days after injection under various conditions. Scale bar: 100 μm.

We performed a histological analysis of the major organs (heart, liver, spleen, lung, and kidney) for the ink@hydrogel combined with DHA group (Figure 5). The results indicated that the synergistic ink@hydrogel and DHA therapy did not cause deep pathological changes in the organs, suggesting that there was no significant histological abnormality in the treatment groups.

## Conclusion

In summary, we designed a newfangled ink@hydrogel system, which can produce a synergistic activity with DHA for the treatment of cancer. The ink in the ink@hydrogel could generate huge heat energy and hyperthermia when under 1064 nm laser irradiation as it possessed good photothermal performance and stability. Furthermore, Hu Kaiwen ink as a an NIR-II photo-thermal material has great maximum permissible exposure (MPE) and a larger penetration depth. Then, Fe^3+^ ions rapidly diffused from the hydrogel to the tumor microenvironment with dissolution of ink@hydrogel and were reduced to Fe^2+^ to promote the breakage of the endoperoxide bridges in the previously-injected DHA. This resulted in the release of free radicals for a potent anti-cancer effect. And the drug release rate can be controlled by changing different condition. *In vitro* and *in vivo* experiments illustrated the great therapeutic effect of DHA-Fe^2+^. To our best knowledge, this is the first report of a hydrogel tumor therapy system that generates a photo-thermal response in the NIR-II window. We envisioned that this special hydrogel treatment way holds great potential in synergistic tumor therapy.

## Data Availability Statement

All datasets generated for this study are included in the article/supplementary material.

## Ethics Statement

The animal study was reviewed and approved by Wuhan University Animal Care Facility.

## Author Contributions

DC and CC performed the experiments, analyzed all the data, drafted all the figures, and prepared the manuscript. CH performed the experiments. TC and ZL conceived, designed the experiments, and revised the manuscript.

### Conflict of Interest

The authors declare that the research was conducted in the absence of any commercial or financial relationships that could be construed as a potential conflict of interest.
